# Multimorbidity in Adult Asylum Seekers: A First Overview

**DOI:** 10.1371/journal.pone.0082671

**Published:** 2013-12-20

**Authors:** Carmen A. Pfortmueller, Manuela Stotz, Gregor Lindner, Thomas Müller, Nicolas Rodondi, Aristomenis K. Exadaktylos

**Affiliations:** 1 University Department of General Internal Medicine, University Hospital and University of Bern, Bern, Switzerland; 2 Division of General Internal Medicine, Baden Cantonal Hospital, Baden, Switzerland; 3 University Hospital of Psychiatry and University of Bern, Bern, Switzerland; 4 University Department of Emergency Medicine, University Hospital and University of Bern, Bern, Switzerland; Iran University of Medical Sciences, Iran (Republic of Islamic)

## Abstract

**Principals:**

Over the last two decades, the total annual number of applications for asylum in the countries of the European Union has increased from 15,000 to more than 300,000 people. The aim of this study was to give a first overview on multimorbidity of adult asylum seekers.

**Methods:**

Our retrospective Swiss single center data analysis examined multimorbidity of adult asylums seekers admitted to our ED between 1 January 2000 and 31 December 2012.

**Results:**

A total of 3170 patients were eligible for the study; they were predominantly male (2392 male, 75.5% versus 778 female, 24.5). The median age of the patients was 28 years (range 28–82). The most common region of origin was Africa (1544, 48.7%), followed by the Middle East (736, 23.6%). 2144 (67.6%) of all patients were not multimorbid. A total of 1183 (37.7%) of our patients were multimorbid. The mean Charlson comorbidity index was 0.25 (SD 1.1, range 0–12). 634 (20%) of all patients sufferem from psychiatric diseases, followed by chronic medical conditions (12.6%, 399) and infectious diseases (4.7%, 150). Overall, 11% (349) of our patients presented as a direct consequence of prior violence. Patients from Sri Lanka/India most often suffered from addictions problems (50/240, 20.8%, p<0.0001). Infectious diseases were most frequent in patients from Africa (6.6%), followed by the Balkans and Eastern Europe/Russia (each 3.8%).

**Conclusion:**

The health care problems of asylum seekers are manifold. More than 60% of the study population assessed in our study did not suffer from more than one disease. Nevertheless a significant percentage of asylum seekers is multimorbid and exhibits underlying psychiatric, infectious or chronic medical conditions despite their young age.

## Introduction

During the past 60 years, there have been more than 200 wars worldwide; these had and still have a profound impact on health, which exceeds the impact of injuries directly attributable to the conflict itself [1]. There are many refugees from war, war-like conditions or political or ethnic oppression [2]. At the end of 2009, there were 43.3 million people worldwide who had been forcibly displaced due to conflict and persecution – the highest number since the mid 1990s [3]. Over the last two decades in the countries of the European Union, the total number of applications for asylum has increased from 15,000 to more than 300,000 people annually [4]. Individuals fleeing persecution have the right to asylum [5]. This most fundamental right was guaranteed by the UN convention in 1951 and was implemented in 1967 [5]. As asylum seekers come from countries with violent conflicts and make a hazardous journey to the country of asylum, their disease profile is strikingly different from that of the native population in the country of application [1]. This is not only due to the violence itself, but is also a consequence of the absence of a functioning health care system in the country of origin [1]. Moreover, the asylum process itself is connected to a long phase of waiting in uncertainty and under unfavorable living conditions, which further exacerbates health care problems [6]. Clinical observations have shown that the detention-like living conditions of asylum seekers cause additional distress by extending the uncertainty and fear generated by past experiences of persecution [7]. According to the Swiss Federal Agency of Immigration, there were 40,670 people in the asylum process in 2011 [8]. New applications increased annually from 19,750 in 2000 to 24,667 in 2012 [8]. There was a striking increase in applications in 2011 - by more than 45%. This was the greatest increase since 2002 [8]. For the duration of the asylum process, the Swiss government provides mandatory basic healthcare coverage [2]. All asylum seekers may register free of charge with a general practitioner [9].

Several articles have addressed political policies concerning asylum seekers, but less has been said about their health problems [4]. Most of the existing studies focus on mental health problems [10]–[13]. Few studies have dealt with multimorbidity of asylum seekers and not a single study has compared the disease profiles of asylum seekers from different regions. As asylum seekers often do not attend a primary care physician emergency department, as they are not used to the concept of primary care prior to specialist treatment [14], emergency department personnel should be educated on the particular disease profile of these patient population and on how to provide more specific care.

We have therefore assessed all emergency department (ED) admissions of asylum seekers within a twelve year period. The aim of this study was to give a first overview on multimorbidity of adult asylum seekers.

## Materials and Methods

### Setting

Our ED is the only Level I ED in central Switzerland, serving about 1.8 million people and treating more than 35,000 cases per year.

### Data Collection and Retrospective Survey

Our retrospective data analysis comprised adult (≥16 years) patients admitted to our emergency department (ED) between 1 January 2000 and 31 December 2012 with the official resident status of an “asylum seeker” or “refugee”. Resident status is routinely assessed by our hospital administration. Patients were identified using the appropriate search string in the patient’s demographic field of our computerized patient database (Qualicare Office, Medical Database Software, Qualidoc AG, Bern, Switzerland). Additionally all records were matched with our hospitals central patients database where all past medical records are stored. Therefore the medical records we reviewed for the present study contained information about all diagnosis a patients ever received in our hospital and not only from the actual emergency department visit. The matching does not depend on out- or inpatient status of a visit. The following clinical data were extracted from medical records: reason for presentation, number of diseases, psychiatric disease (if/type) and infectiological diseases (if/type) were evaluated. Additionally chronic medial symptom complexes with unclear origin such as a history of inappetence, a history of sleep disorder, a history of chronic dizziness, chronic musculoskeletal pain, chronic gastrointestinal problems, chronic headaches and hospitalization rate were also assessed. Demographic data, such as gender and age, were also assessed. After reviewing the literature, we found no consistent definition or approach to assess multimorbidity [15]. Others have assessed multimorbidity ranging from 7 different diseases [16] to 46 [17]. We therefore derived a new list of diseases based on the largest study by Higashi *et al* [18] and the Charlson index [19]. Additionally, we included psychiatric conditions (e.g. schizophrenia) as an important type of disease [20] based on a consensus of the above mentioned references and between the authors. The final list contains 18 important diseases for ambulatory medicine, see table 1.

**Table 1:** List of Diseases.

Myocardial Infarction
Heart Failure
Peripheral Vascular Disease
Cerebrovascular Disease
Dementia
COPD
Connective Tissue Disease
Peptic Ulcer Disease
Diabetes Mellitus
Moderate to Severe Chronic Kidney Disease
Malignant Tumor (solid or non-solid)
Liver Disease
Psychiatric Disease (any type)
Osteoporosis
Hypertension
Dyslipidemia
Asthma
Chronic Infectious Disease

The severity of multimorbidity was assessed by the Charlson comorbidity index [19]. The Charlson comorbidity index is calculated based on points given to predefined medical conditions such as a history of myocardial infarction, active neoplastic disease ect. [19]. Additionally points are given for the age of the patient [19]. Chronic medical symptom complexes with unclear origin were defined as self-reported somatic health problems of more than one month duration. These included: history of inappetence, history of sleep disorder, history of chronic dizziness, chronic musculoskeletal pain, chronic gastrointestinal problems and chronic headaches. Infectious dieseases were defined as human retrovirus infection (HIV), hepatitis B and C, syphilis, gonorrhea, parasitic infections and tuberculosis. All medical records were reviewed by two specialists in internal medicine and a specialist in emergency medicine. Records of patients with psychiatric disorders were discussed with a psychiatrist. Nationalities were grouped into geographical regions (Africa, Asia, Southern/Middle America, Balkan countries, Middle East, Eastern Europe/Russia, Sri Lanka/India). The geographical definition of Middle East varies [21], we included patients from the following countries: Afghanistan, Armenia, Asarbaidshan, Bahrain, Egypt, Georgia, Irac, Iran, Israel, Yemen, Jordan, Kazakhstan, Kuwait, Lebanon, Pakistan, Syria, Saudi Arabia, Quatar, Turkey, Turkmenistan, Uzbekistan and United Arab Republic. Patients with incomplete medical records (rudimentary history, lack of diagnosis and systemic history), duplicated records and recurrent presentations, were excluded from the analysis.

### Statistical Analysis

All statistical analyses were performed with the SPSS 20.0 Statistical Analysis program (SPSS Inc; Chicago, IL). The data were summarised using descriptive statistics (means and standard deviation or medians and range as appropriate, counts and percentages). Differences in characteristics and regional origin between patients were tested using chi-squared tests for categorical variables and Kruskal-Wallis ANOVA for interval and ordinal variables. Post-hoc testing was performed using the Mann-Whitney U test. All p values were two tailed and at a level of significance of 0.05.

### Ethical Considerations

The study was approved by the Ethics Committee of the Canton of Bern, Switzerland. Individual patient consent was not obtained, it was waived by the Ethics Committee.

## Results

**Figure 1:**
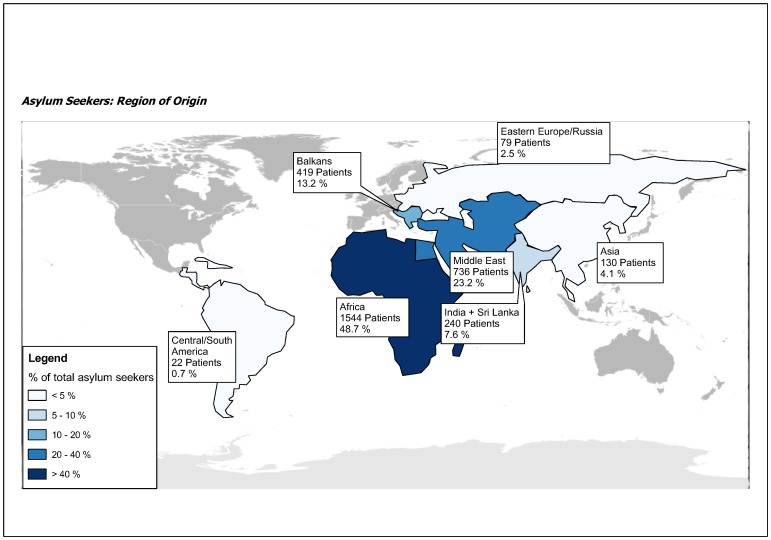
Overview on geographical origin.

**Figure 2:**
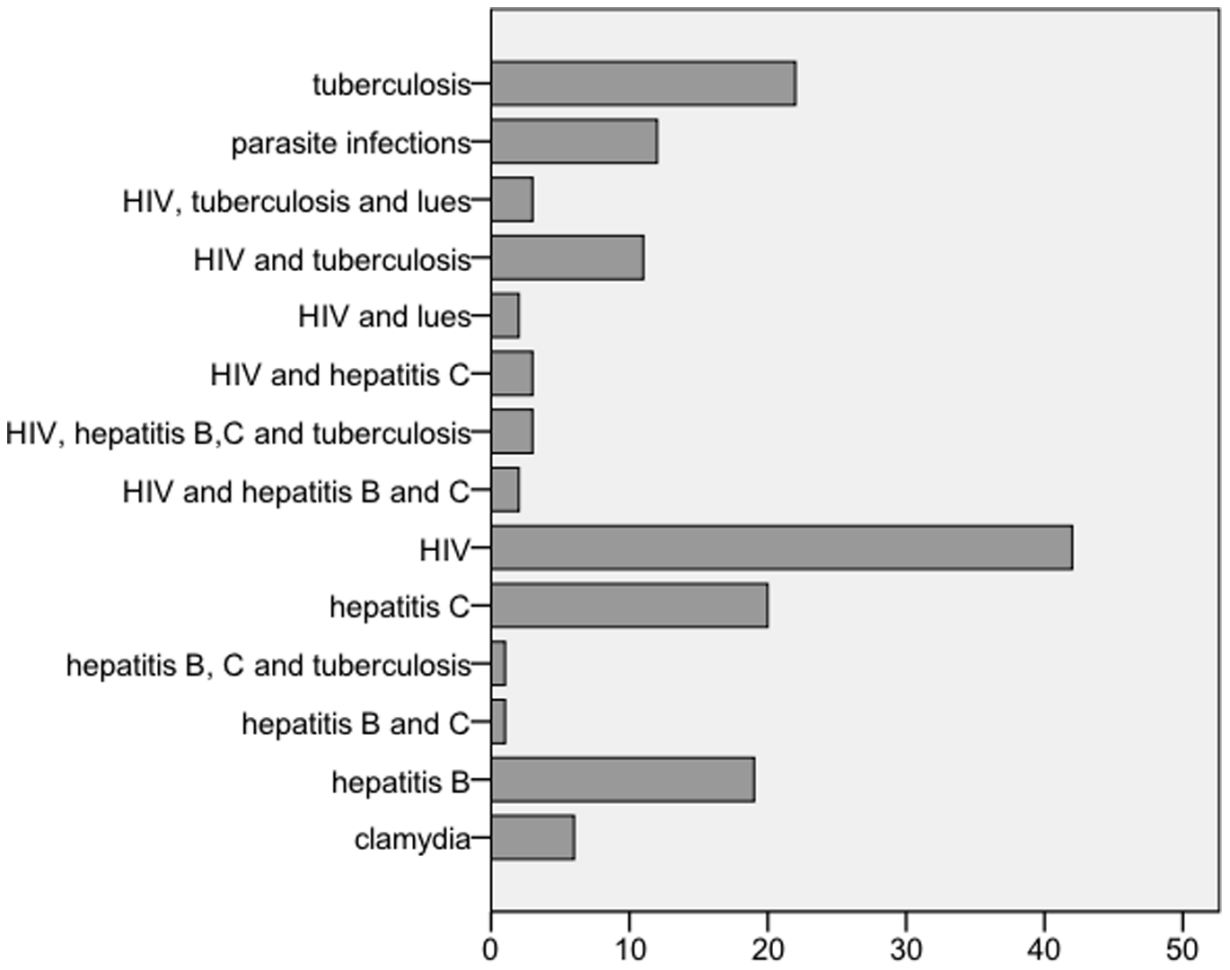
Overview of infectious comorbidities (absolute numbers).

**Figure 3:**
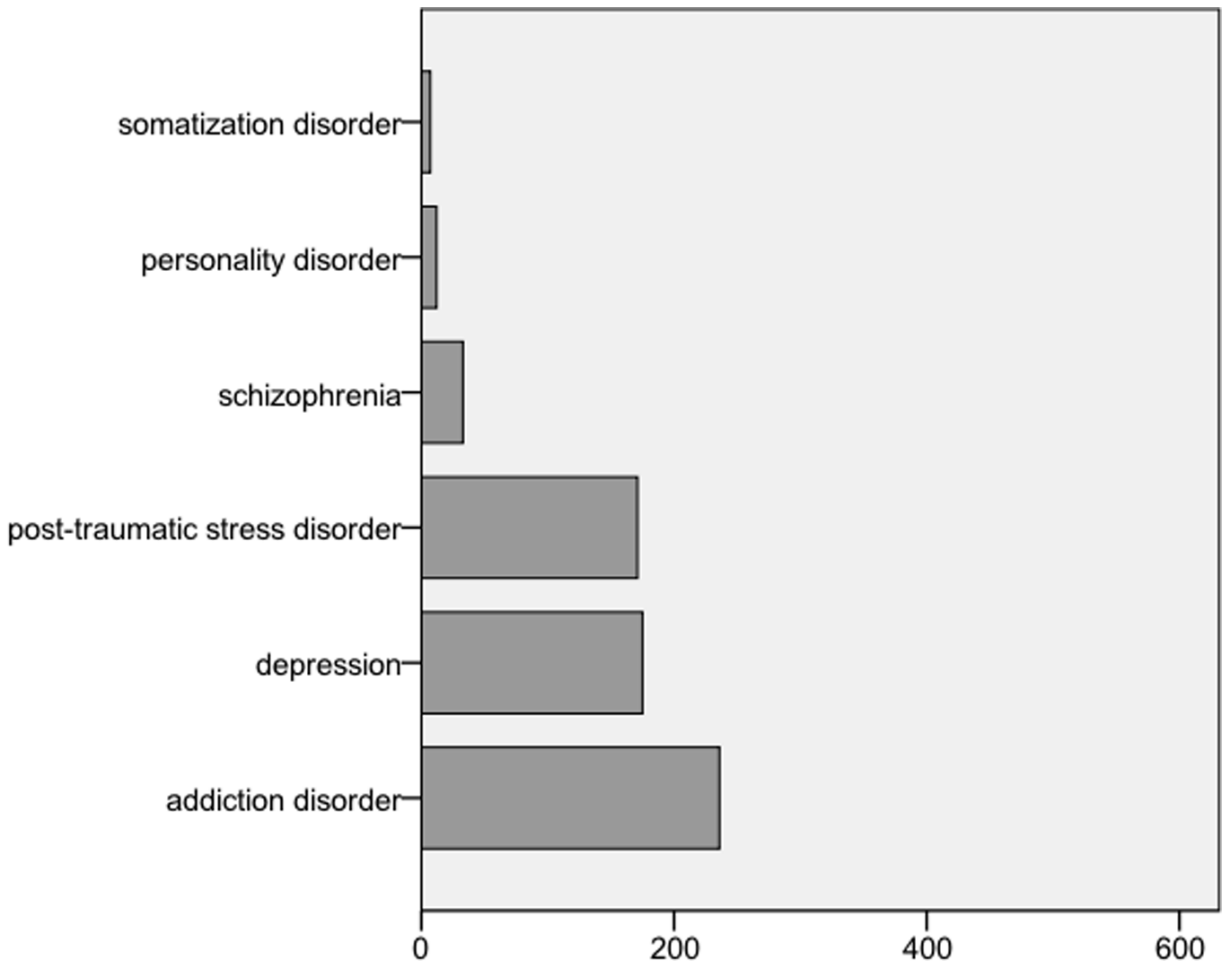
Overview of psychiatric comorbidities (absolute numbers).

A total of 3170 patients were eligible for the study. 98.1% (3107) of these patients were asylum seekers, 1.9% (62) refugees. Patient characteristics are described in table 1. The median age of the patients was 28 years (range 28–82). The study population was predominantly male (2392, 75.5% versus 778, 24.5%). Males were significantly younger than females (mean 29.4, SD 9.9 versus mean 34.2, SD 12.1, p<0.0001). The most common region of origin was Africa (1544, 48.7%), followed by the Middle East (736, 23.6%). For an overview on region of origin see figure 1. Presentation was most often for surgical reasons, in 1557 cases (48.7%), followed by medical reasons (1259, 39.7%) and psychiatric causes (306, 9.7%). Overall, 11% (349) of our patients presented as a direct consequence of prior violence. A total of 1183 (37.7%) of our patients were multimorbid. The mean disease count was 0.58 (SD 1.2, range 0.9) and the mean Charlson comorbidity index was 0.25 (SD 1.1, range 0–12). 634 (20%) of all patients suffered from a psychiatric disease, followed by chronic medical symptom complexes of unclear origin (12.6%, 399) and infectious diseases (4.7%, 150). Figures 2 and 3 display an overview of the types of psychiatric and infectious diseases. 1217 (38.4%) of the patients were admitted to hospital. Among chronic medical conditions, chronic gastrointestinal problems were most common (152, 38.1%), followed by chronic musculoskeletal pain (124, 31.1%), chronic headaches (89, 22.3%), sleeping disorders (72, 18.0%), inappetence (32, 8.0%) and chronic dizziness (14, 3.5%).

Table 2 and 3 present an overview of reasons for admission and multimorbidity in relation to geographical origin. Males were predominant for all geographical regions. The male to female ratio was smallest in patients originating from the Balkans (p<0.0001). Patients originating from Sri Lanka or India were significantly older than all other patients, but patients from Asia were significantly younger than all other patients (both p<0.0001). Patients originating from Sri Lanka/India exhibited significantly higher disease counts than others, whereas patients originating from Asia had a significantly lower disease count than others (both p<0.0001). The Charlson comorbidity index was significantly higher for patients originating from Sri Lanka or India than for other patients (p<0.0001). Patients originating from Southern America and Sri Lanka/India most often exhibited a psychiatric diseases (22.7 versus 17.9%, p<0.0001). Patients from Sri Lanka/India most often suffered from addictions problems (50/240, 20.8%, p<0.0001). Patients originating from Africa (6.6%), followed by the Balkans and Eastern Europe/Russia (each 3.8%), most often suffered from infectious diseases (p<0.0001). HIV was most common in African asylum seekers (42, 2.7%); patients originating from the Balkans suffered most often from hepatitis B (16, 2.1%) and patients from Eastern Europe/Russia most often suffered from tuberculosis (2, 2.5%) (all p<0.0001). No subpopulation predominance was found for hepatitis C.

**Table 2:** Patient characteristics.

	N	%
N	3170	100
male	2392	75.5
female	778	24.5
age (median, range)	28 (16–82)	
**Nationality**		
Africa	1544	48.7
Balkans	419	13.2
Eastern Europe/Russia	79	2.5
India+Sri Lanka	240	7.6
Asia	130	4.1
Central/South America	22	0.7
Middle East	736	23.2
**Reason for Presentation**		
medical	1259	39.7
surgical	1557	49.1
gynecological	15	0.5
ear nose throat	33	1
psychiatric	306	9.7
**Count of Diseases**(mean, SD, range)	0.58 (1.2, 0–9)	
**Charlson Comorbidity Index**(mean, SD)	0.25 (1.1, 0–12)	
**Subgroups**		
psychiatric	634	20.0
infectious	150	4.7
chronic medical symptom complexeswith unclear origin	399	12.6
**Hospitalization rate**	1217	38.4

**Table 3:** Overview of reasons for admission and multimorbidity in relation to geographical origin.

	Africa	Balkans	EasternEurope/Russia	Sri Lanka/India	Asia	Middle/SouthAmerica	MiddleEast	p value
**Total**	1544	419	79	240	130	22	736	0.0001
male (%)	1157 (74.9)	266 (63.4)	51 (64.5)	178 (74.1)	105	18 (80.7)	617 (83.8)	0.0001
female (%)	387 (25.1)	153 (36.6)	28 (25.1)	62 (25.9)	25	4 (19.3)	119 (16.2)	0.0001
age (median, range)	27 (16–76)	29 (16–81)	29 (16–62)	36 (16–82)	22 (16–60)	30 (17–67)	30 (16.–73)	0.0001
**Reason for Admission**								
medical	641 (41.5)	159 (37.9)	29 (36.7)	103 (42.9)	41 (31.1)	10 (45.5)	276 (37.5)	0.152
surgical	760(49.2)	200 (47.7)	32 (40.4)	107 (42.6)	81 (62.3)	11 (50.0)	366 (49.7)	0.032
psychiatric	124 (8.0)	51 (12.2)	10 (12.7)	28 (11.7)	4 (3.1)	1 (4.5)	88 (12.0)	0.0001
others	19 (1.2)	9 (2.1)	8 (10.1)	2 (0.8)	4 (3.1)	0 (0)	6 (0.8)	0.0001
**Count of Disease**(total (mean, range)	1 (0–9)	1 (0–8)	1 (0–5)	1 (0–7)	0 (0–2)	1 (0–3)	1 (0–9)	0.0001
**Charlson Co-Morbidity** **Index** (mean, range)	0 (0–11)	0 (0–9)	0 (0–7)	1 (0–12)	0 (0–2)	0 (0–2)	0 (0–11)	0.0001
**Subgroups** (number, %)								
psychiatric	136 (8.8)	71 (16.9)	12 (16.5)	43 (17.9)	6 (4.6)	5 (22.7)	117 (15.9)	0.0001
infectious	102 (6.6)	16 (3.8)	3 (3.8)	2 (0.8)	8 (6.2)	0 (0)	19 (2.6)	0.0001
chronic medical symptomcomplexes with unclear origin	186 (45.9)	54 (13.5)	5 (1.3)	40 (10.0)	12 (3.0)	1 (0.3)	104 (26.1)	0.0001

## Discussion

Among 3170 admissions of asylum seekers to our emergency center, we found that about 40% of asylum seekers suffered from multiple diseases, of which psychiatric diseases were most common.

In our study patients were quite young with a median age below 30 years. To the best of our knowledge this is the first study examining multimorbidity in asylum seekers. Therefore we do not have any figures to compare our results to. But eventhough our patients were quite young they suffered from multiple diseases in significant percentage. The high percentages of psychiatric diseases is not surprising, as asylum seekers have often had a traumatic history, leading to higher rates of depression, anxiety and posttraumatic stress disorder than in the general population [14], [22]–[24]. Additionally, post-migration resettlement stressors, such as social isolation, financial uncertainty and unfavorable living conditions also adversely affect refugees’ mental health [14].

Astonishingly chronic medical symptom complexes are even more common than infectious diseases amongst asylum seekers [14]. According to a review by Eckstein et al are chronic medical symptom complexes such as musculoskeletal and gastrointestinal pain, and chronic headaches common amongst asylum seekers [14], a finding that we confirm. These syndromes most often have both physical and psychological origins. The contributory factors include past physical trauma, hard physical labor in an often high risk job with no appropriate training, difficult housing conditions (such as sleeping on the floor), as well as past psychological trauma [12]–[14]. The organic causes of the pain are often difficult to identify, despite extensive workups [14], [23]. 10–35% of asylum seekers from former Yugoslavia with chronic medical complaints have been reported to suffer from a somatization disorder [13]. There have also been repeated reports that somatic complaints have a central role in the presentation of posttraumatic stress in Somali refugees [12]. Thus, once a somatic cause for these symptoms has been excluded by a careful examination, these patients should be assessed for posttraumatic stress disorder.

Several studies have showed that the rate of infectious diseases, such as hepatitis and HIV, is higher in asylum seekers than in the native population in the country of application [25], [26]. Asylum seekers constitute a special social group in a geographical area, as they often live under conditions that facilitate the spread of infectious diseases [25], [26]. Moreover, the infectious disease profile in their country of origin may be strikingly different than in the country of application [25], [26]. In our study, the prevalence of hepatitis B was most common in asylum seekers from the Balkans. Roussos et al found a similar result in their study on the prevalence of hepatitis B and C markers among refugees in Athens [25]. They screened 130 asylum seekers for hepatitis B and C and found a prevalence of anti-HBsAG of 15.4% in Balkan asylum seekers, a figure that is remarkably higher than in our study [25]. The higher prevalence found by Roussos et al is most likely related to the systematic screening they performed, whereas in our study the prevalence of hepatitis B was retrospectively assessed by narrative comments. We did not find any difference in the prevalence of hepatitis C between the different geographical subgroups, a finding that is also confirmed by Roussos et al [25]. Tafuri et al screened mainly (96.4%) African asymptomatic asylum seekers in southern Italy for hepatitis B and C, HIV and syphilis and found that 1.5% of their study population was HIV positive [26]. In our study, 2.7% of the African asylum seekers were HIV positive. There may be several reasons for this difference Firstly, the Italian study excluded symptomatic individuals. Secondly, as we are a tertiary hospital with a 24/7 infectiological service on-call, patients with already diagnosed HIV infection will most probably present to our ED with HIV-related complications, so that our figures may be higher than in other Swiss hospitals. As the prevalence of hepatitis and other infections is high amongst asylum seekers, constant surveillance would seem to be essential to monitor the prevalence of these infections. Their transmission could then be controlled through counseling and vaccination programs wherever possible [26].

In our study, Sri Lankan/Indian asylum seekers were older and had a higher disease count than others. In particular, psychiatric and addiction problems were more common amongst Sri Lankan/Indian asylum seekers in the present study. The Sri Lankan conflict lasted 26 years and resulted in 800,000 people being displaced [11]. According to Brune et al, addiction problems are generally very common amongst asylum seekers [27]. According to a review by Rajapakse et al on the characteristics of non-fatal self-poisoning in Sri Lanka, the rate of non-fatal self-poisoning - such as alcohol or drug abuse - is strikingly higher in Sri Lanka than in other developing or first world countries [28]. One possible explanation for this may be that self-poisoning may be considered an acceptable way of coping with stress and conflict in Sri Lanka [28]. The collectivistic rather than individualistic nature of Sri Lankan society, the hierarchical framework in which open confrontation is discouraged, and the strong sense of shame, are all causes of self-poisoning in response to interpersonal conflict [28]. This may explain why psychiatric diseases and especially substance abuse is more common in Sri Lankan/Indian refugees than in others.

### Limitations

This study has to be interpreted with some caution as it has specific limitations. As this was a retrospective study, no standardized general and systemic medical history was taken. Therefore it is possible that our figures on disease count and type, especially psychiatric and infectious diseases, are underestimated. Due to the retrospective design, chronic medical conditions may not have been detected, if not reported by the patients themselves and if patients were treated in another hospital or by a primary care physician. This study provides a first overview on multimorbidity in adult asylum seekers and further prospective studies should be conducted. Additionally an emergency department where patients often are treated with limited time and personal resources may not be the most favorable place to study multimorbidity in asylum seekers. Therefore our figures may underestimate the actual multimorbidity in this population. False assessment of nationality and geographic region cannot be excluded. Asylum seekers and refugees are different population this may influence age distribution, general health and count/type of disease. Eventhough refugees account for less than 2% of our study population our results may be biased. Furthermore, as this is a single center study, external validity is not given. Additionally, we only assessed patients with the status of “asylum seeker/refugee”. We did not assess patients with a residents permit but from outside Switzerland. Therefore our data does not represent an analysis of the health care problems of persons of a specific nationality. Moreover, we cannot provide any information on the medical conditions of children, as children are treated in a different ED within our hospital.

## Conclusion

The health care problems of asylum seekers are manifold. More than 60% of the study population assessed in our study did not suffer from more than one disease. Nevertheless a significant percentage of asylum seekers is multimorbid and exhibits underlying psychiatric, infectious or chronic medical conditions despite their young age. These highly follow a geographical pattern. Infectious diseases are more common amongst asylum seekers than amongst the residents of the country of application.

This study provides a first overview on multimorbidity in adult asylum seekers. Further prospective multicenter studies should be conducted to further assess multimorbidity in asylum seekers.
